# Efficacy and safety of traditional Chinese medicinal enemas for treatment of chronic renal failure: A protocol for systematic review and meta-analysis

**DOI:** 10.1097/MD.0000000000023002

**Published:** 2020-10-30

**Authors:** Lihua Wu, Yating Wang, Yu Liu, Ling Wu, Dan Cheng, Ting Jiang, Bo Qu, Hongmei Lu, Ju Yang, Anqi Tang, Mingquan Li

**Affiliations:** Department of Nephrology, Hospital of Chengdu University of Traditional Chinese Medicine, Chengdu, Sichuan, PR China.

**Keywords:** chronic renal failure, meta-analysis, protocol, systematic review, traditional Chinese medicinal enemas

## Abstract

**Background:**

Chronic renal failure (CRF) is a common kidney disease characterized by a slow and progressive decline in kidney function. Clinical practice suggests that traditional Chinese medicinal enemas have a therapeutic effect on CRF. To assess the therapeutic efficacy and safety of traditional Chinese medicinal enemas in treating CRF, we created a protocol for a systematic review to inform future clinical applications.

**Methods:**

We completed a literature search of all clinical randomized controlled trials evaluating traditional Chinese medicinal enemas on CRF in the following five English and four Chinese databases completed before August 2020: Medline, EMBASE, Web of Science, Cochrane Central Register of Controlled Trials (CENTRAL), Cochrane Library database, Chinese National Knowledge Infrastructure (CNKI), WANFANE Database, Chinese Scientific and Technological Periodical Database (VIP) and Chinese Biomedical Database (CBM). The primary outcomes evaluated blood urea nitrogen levels, uric acid levels, endogenous creatinine clearance rate, and serum creatinine, and the secondary outcomes included clinical efficacy and adverse effects of treatment. Two independent researchers performed data extraction and quality assessment. RevMan5.3 software was used to assess data quality and bias. This protocol was conducted according to the Preferred Reporting Item for Systematic Review and Meta-analysis Protocol (PRISMA-P) statement.

**Results:**

This study will provide a rational synthesis of current evidence for traditional Chinese medicinal enemas for the treatment of CRF.

**Conclusion:**

This study presents evidence on whether traditional Chinese medicinal enemas are an effective and safe intervention for CRF patients.

**Registration number:**

INPLASY202080052

## 1 Introduction

Chronic kidney disease (CKD) is a global public health concern^[1]^ due to high prevalence globally,^[2]^ adverse health effects,^[3]^ and high costs for both patients and the healthcare system.^[1]^ As a clinical manifestation of advanced CKD, chronic renal failure (CRF) is characterized by a slow and progressive decline in kidney function. Recently, the morbidity and mortality of CRF have risen significantly, resulting in a heavy economic burden worldwide. The efficacy of CRF treatment has improved remarkably with the development of renal replacement therapies (RRT) such as hemodialysis, hemodiafiltration, peritoneal dialysis, and kidney transplantation. However, the mortality rate of patients using RRT is very high, and in developing countries, RRT is performed in less than 25% of CRF patients.^[4]^ Thus, delaying CKD progression would benefit patients, social economics, and healthcare systems alike.

Advanced CKD may progress to permanent kidney failure. One of the risk factors for this is an imbalanced intestinal microbiota, characterized by a decline of probiotics and an increase in opportunistic pathogens, such as urease-related microbes, endotoxin-related microbes and toxin-related microbes which generate uremic toxins.^[5,6]^ In accordance with the “the gut-kidney axis” hypothesis^[7]^ and “the chronic kidney disease-colonic axis”,^[8]^ dysregulation of intestinal microbiota irritates renal tissue through increasing uremic toxins, causing systemic micro-inflammation and tissue damage. Animal experiments and preliminary clinical trials suggest that a variety of single Chinese herbal medicines (CHM) administered either orally or via enema can regulate intestinal flora dysbiosis,^[9,10]^ protect the intestinal epithelial barrier,^[11,12]^ decrease uremic toxin accumulation, and postpone CKD progression.^[13,14]^

Among non-dialysis treatment methods, Traditional Chinese Medicine (TCM) is increasingly demonstrating unique advantages. The TCM enema method is based on invigorating the kidney and removing turbidity. The enema fluid accelerates the elimination of metabolic waste through the intestine and reduces its damage to vital organs. Currently, Chinese medicine enema has been widely used in the treatment of CRF. To objectively evaluate the effect of Chinese medicine enema in treating CRF, we performed an evidence-based medical system evaluation by comprehensively collecting data referencing the use of traditional Chinese medicine enema in clinical treatment of CRF. We reviewed clinical trials to evaluate the objective efficacy of CRF treatment with TCM enema to provide reliable evidence for informed clinical practice.

## 2 Methods

### 2.1 Registration

The protocol of this systematic review has been registered on INPLASY.COM (registration number: INPLASY202080052). The program will be based on the Preferred Reporting Items for Systematic Reviews and Meta-analysis Protocols (PRISMA-P) 2015 statement.^[15]^

### 2.2 Eligibility criteria

#### 2.2.1 Study type

All available randomized controlled trials (RCTs) on traditional Chinese medicinal enemas for CRF were included. Non-RCTs and uncontrolled clinical trials were excluded. There was no unified requirement for blinding and language.

#### 2.2.2 Participants

##### 2.2.2.1 Diagnostic criteria^[16]^

- Endogenous creatinine clearance rate (Ccr) < 80 mL/min;
- Serum creatinine (Scr) >133 umol/L;
- Having a history of CKD or kidney-involved systemic disease.

##### 2.2.2.2 Inclusion criteria

- Patients meeting the above diagnostic criteria;
- Non-dialysis patients under stable condition were enrolled for reversible factor observation such as infection, pre-heart failure, and hypovolemia;
- Patients must be ≥ 18 years old regardless of their race, sex, economic or education status.

##### 2.2.2.3 Exclusion criteria

- Pregnant or lactating female;
- Patients with severe primary diseases such as brain, heart, liver, and hematopoietic system diseases;
- Patients undergoing psychiatric treatment who cannot implement the treatment;
- Under 18 years old.
- Repeatedly published literature;
- Literatures without full text or lacking original data.

#### 2.2.3 Interventions and comparators

Other conventional treatments could be combined for patients in the intervention group; in the control group, only conventional treatments were included.

#### 2.2.4 Outcome measures

##### 2.2.4.1 Primary outcomes

The primary outcomes assessed include blood urea nitrogen levels, uric acid levels, Ccr, and Scr.

##### 2.2.4.2 Secondary outcomes

The secondary outcomes include clinical efficacy and adverse health events. The clinical efficacy refers to the guiding principles for clinical research of new Chinese medicines and is determined by symptomatic improvement upon treatment. Treatment was considered markedly effective when the following criteria were met: reduced or resolved clinical symptoms, Ccr increased by ≥20% or Scr decreased by ≥20%. Treatment was considered effective when: clinical symptoms reduced or disappeared, Ccr increased by ≥10% or Scr decreased by ≥10%. Ineffective treatment was determined as clinical symptoms that do not resolve, a Ccr increase of ≤10% or a Scr decrease of ≤10%.

### 2.3 Electronics searches

RCTs published until August 2020 were consulted from the following five English and four Chinese databases: Medline, EMBASE, Web of Science, Cochrane Central Register of Controlled Trials (CENTRAL), Chinese National Knowledge Infrastructure (CNKI), WANFANE Database, Chinese Scientific and Technological Periodical Database (VIP), Chinese Biomedical Database (CBM), and the Cochrane Library database. To avoid missing eligible studies, some literature was searched for manually. Ongoing trials were searched on the international clinical trial registry (http://clinicaltrials.gov/) and the Chinese clinical trial registry (http://www.chictr.org.cn/). Searches were conducted in English and Chinese. Additionally, clinical trials were obtained by checking the reference lists of relevant literature, conference proceedings, and gray literature. The search keywords were used alone or in various combinations. The searching strategy of PubMed is shown below:

#1 Kidney Failure, Chronic [MeSH]
#2 “End Stage Kidney Disease” [Title/Abstract] OR “Chronic Kidney Failure” [Title/Abstract] OR “End-Stage Renal Disease” [Title/Abstract] OR “End Stage Renal Failure” [Title/Abstract] OR “Chronic Renal Failure” [Title/Abstract] OR “ESRD” [Title/Abstract]
#3 #1 OR #2
#4 Medicine, Chinese Traditional [MeSH]
#5 “End Stage Kidney Disease” [Title/Abstract] OR “Traditional Chinese Medicine” [Title/Abstract] OR “Chung I Hsueh” [Title/Abstract] OR “Zhong Yi Xue” [Title/Abstract] OR “Traditional Tongue Diagnosis” [Title/Abstract] OR “Traditional Tongue Assessment” [Title/Abstract] OR “Traditional Tongue Assessmen∗”
#6 #4 OR #5
#7 Enema [MeSH]
#8 Enema∗ [Title/Abstract]
#9 #7 OR #8
#10 #3 AND #6 AND #9

### 2.4 Study selection and data extraction

**Figure 1:**
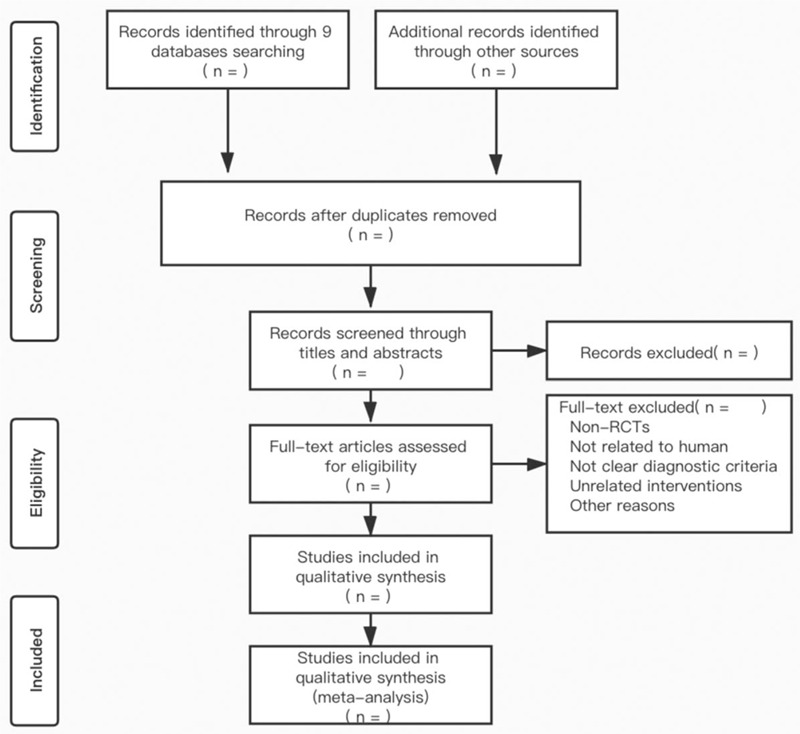
Flow diagram of the trial selection process.

Literature citation was managed using EndNote X9. After removing duplicate studies, two reviewers (BQ and HL) evaluated the title and abstract of each article independently. Upon reviewing the full articles, further screens were performed to identify qualified studies. A third reviewer (Mingquan Li) was consulted when there was disagreement regarding study inclusion criteria. The selection process is summarized in a PRISMA flowchart (Fig. 1).

A standardized table of databases was used to extract relevant information, including title, first author, year of publication, sample size, country of publication, subjects’ age and gender, intervention, treatment duration, outcomes, and adverse events. Two reviewers (YL and LW) independently performed the data extraction, and to ensure accuracy, all extracted data were cross-checked by both reviewers. When necessary, a third reviewer (ML) made decisions on data extraction.

### 2.5 Assessment of risk of bias

The “risk of bias” tool from the Cochrane Handbook for Systematic Reviews of Interventions was used for quality assessment for each publication.^[17]^ The handbook assesses six qualities for bias risk, including random sequence generation, allocation concealment, blind controls, data outcome integrity, selective outcome reporting, along with other risks. Two reviewers (TJ and LW) categorized each study as “high risk”, “low risk”, or “unclear risk” in accordance with these 6 factors. The corresponding authors were consulted for detailed data when there was unclarity. The third reviewer (ML) resolved all disagreements.

### 2.6 Statistical analysis

#### 2.6.1 Meta-analysis

Review Manager 5.3, a statistical software provided by the Cochrane collaboration, was used to perform the meta-analysis. Continuous data were expressed as the mean difference (MD) with a 95% confidence interval (CI). Count data were expressed as relative risk ratios (RR) with a 95% CI. For all analyses, a *P* value < .05 represented statistical significance. Potential heterogeneity was reflected by the ***χ***^2^ and ***I***^2^ test.

#### 2.6.2 Data synthesis

When there was heterogeneity between studies (*P* ≤ .01, ***I***^2^ ≥ 50%), the random-effect model was applied for combination analysis, subgroup analysis, or sensitivity analysis depending on the source of heterogeneity. When the heterogeneity test revealed no heterogeneity between groups (*P* > .01, ***I***^2^ < 50%), the fixed-effect model was applied for combination analysis.

#### 2.6.3 Dealing with missing data protocol

When studies lacked relevant information, reviewers (JY and AT) contacted the original investigator to obtain the missing data. The intentional analyses and sensitivity analyses were conducted if the missing data could not be acquired.^[18]^

#### 2.6.4 Assessment of heterogeneity

To assess statistical heterogeneity, a standard ***χ***^2^ test with a significance level of *P* < .1 was applied. Studies were not considered heterogeneous when the ***I***^2^ value was < 50%.

#### 2.6.5 Subgroup analysis

To explain heterogeneity, subgroup analyses were performed when possible. Factors including different CHM dosages, intervention forms, and outcome measures were considered.

#### 2.6.6 Sensitivity analysis

Sensitivity analysis was performed to assess the stability of results when heterogeneity was notably different from the methodological quality of enrolled studies. The impact of methodological quality, missing data and sample sizes were evaluated. After removing studies with low-quality methodologies, the analysis was repeated. If quantitative synthesis was not suitable, a comprehensive narrative summarized the characteristics and findings of the studies and investigated the relationship within or between studies.

#### 2.6.7 Publication bias

Funnel plots were applied to assess potential publication bias if more than 10 studies were enrolled. Egger regression tests were performed to identify asymmetry in the funnel plots.^[19]^

#### 2.6.8 Evidence quality evaluation

The evidence quality of the main results was evaluated by GRADE (the Grading of Recommendations Assessment, Development, and Evaluation) approach.^[20]^ Five items were investigated: design limitations, inaccuracies, inconsistencies, indirections, and publication biases. There were 4 levels of assessment: extra-low, low, medium, and high quality.

## 3 Discussion

CKD is a major public health threat that increases the risk of ESKD, cardiovascular disease, and other complications.^[21,22]^ Modern medicine has adopted a comprehensive treatment protocol for CRF, including health education, exercise and diet management, blood sugar control, lipid and blood pressure regulation, RRT therapy, among other treatment methods.^[23]^ So far, there is no effective western medicine to prevent kidney damage in CRF patients. CHM has a long history of effective treatment for kidney disease. The Chinese medicine enema fluid accelerates metabolic waste elimination from the body through the intestine thereby reducing organ damage throughout the body. Therefore, we conducted a systematic review and meta-analysis of enrolled studies that use traditional Chinese medicinal enemas on CRF, to provide evidence of its efficacy.

Certain limitations should be recognized. Some studies operated with small sample sizes. Also, some major methodological flaws exist, such as the design of RCTs is not rigorous. Despite these shortcomings, traditional Chinese medicinal enemas should have a beneficial effect on CRF patients. However, further rigorous studies are needed.

## 4 Ethics and dissemination

Since the data presented here come from published literature and are not associated with patient privacy, ethical approval is not required. The results of this systematic review will be disseminated in a peer-reviewed journal and in conference presentations.

## Author contributions

**Conceptualization:** Lihua Wu, Yating Wang.

**Data curation:** Yu Liu, Ling Wu.

**Formal analysis:** Lihua Wu, Dan Cheng.

**Project administration**: Ting Jiang, Bo Qu.

**Resources:** Mingquan Li.

**Software:** Lihua Wu, Hongmei Lu.

**Visualization:** Ju Yang, Anqi Tang.

**Writing – original draft**: Lihua Wu.

**Writing – review & editing**: Mingquan Li.
